# Spatial analysis of malaria incidence at the village level in areas with unstable transmission in Ethiopia

**DOI:** 10.1186/1476-072X-8-5

**Published:** 2009-01-26

**Authors:** Asnakew K Yeshiwondim, Sucharita Gopal, Afework T Hailemariam, Dereje O Dengela, Hrishikesh P Patel

**Affiliations:** 1 Boston University, Department of Geography and Environment, 675 Commonwealth Ave., Boston, MA 02215, USA; 2 Federal Ministry of Health, PO Box: 1234, Addis Ababa, Ethiopia

## Abstract

**Background:**

Malaria is the leading cause of morbidity and mortality in Ethiopia, accounting for over five million cases and thousands of deaths annually. The risks of morbidity and mortality associated with malaria are characterized by spatial and temporal variation across the country. This study examines the spatial and temporal patterns of malaria transmission at the local level and implements a risk mapping tool to aid in monitoring and disease control activities.

**Methods:**

In this study, we examine the global and local patterns of malaria distribution in 543 villages in East Shoa, central Ethiopia using individual-level morbidity data collected from six laboratory and treatment centers between September 2002 and August 2006.

**Results:**

Statistical analysis of malaria incidence by sex, age, and village through time reveal the presence of significant spatio-temporal variations. Poisson regression analysis shows a decrease in malaria incidence with increasing age. A significant difference in the malaria incidence density ratio (IDRs) is detected in males but not in females. A significant decrease in the malaria IDRs with increasing age is captured by a quadratic model. Local spatial statistics reveals clustering or hot spots within a 5 and 10 km distance of most villages in the study area. In addition, there are temporal variations in malaria incidence.

**Conclusion:**

Malaria incidence varies according to gender and age, with males age 5 and above showing a statistically higher incidence. Significant local clustering of malaria incidence occurs between pairs of villages within 1–10 km distance lags. Malaria incidence was higher in 2002–2003 than in other periods of observation. Malaria hot spots are displayed as risk maps that are useful for monitoring and spatial targeting of prevention and control measures against the disease.

## Background

Malaria is a major public health problem in Africa with over 200 million clinical episodes and nearly one million deaths occurring annually [1,2]. However, the risks of morbidity and mortality associated with malaria, particularly in semi-arid and highland regions, vary spatially and temporally [3-5]. In semi-arid and highland regions of Africa, malaria is unstable and epidemic malaria is a common problem, causing an estimated 12.74 million clinical episodes and 155,000–330,000 deaths annually [4-6]. In Ethiopia, malaria is the leading cause of morbidity and mortality. About 70% of the population (Approximately 52 million people) is estimated to be at risk for malaria infection each year. Health facilities in the country [7] report over five million malaria cases and thousands of deaths across all age groups. Rates of morbidity and mortality dramatically increase (5–6 fold) during epidemic years that recur at irregular intervals of 5–7 years [4,7,8]. Transmission usually occurs in about three-quarters of the country, below 2000 m altitude (but sometimes up to 2500 m). However, the levels of malaria risk and transmission intensity exhibit significant spatial and temporal variability related to variations in climate, altitude, topography, and human settlement pattern [9-13]. The spatial and temporal patterns of malaria transmission at the local level (fixed spatial scale) in semi-arid and highland regions in Africa, and particularly in Ethiopia, have not been well investigated or accurately defined. Such research is needed in developing dynamic and area-specific risk maps to identify locations and populations at highest risk for appropriate planning and implementation of targeted and epidemiologically sound preventive and control measures against the disease.

The existing malaria risk maps have limited operational use to support programmatic activities since they were produced at coarse spatial scales, (at continental and country levels). They are largely based on expert opinion [14], on climate based models [15,16], and specific geo-referenced point prevalence data [17-20]. Other attempts to produce similar risk maps are based on entomological parameters [21,22] that are not fully validated. In addition, researchers have investigated the spatial pattern of malaria distribution at household and community levels in specific sites in relation to proximity of mosquito breeding habitats [23-28]. Households located within 2–3 km of breeding habitats exhibit higher clustering of malaria cases than others [23]. These studies have focused on entomological parameters and population prevalence surveys, which demand skilled personnel and financial resources that may not be readily available.

In this context, geographical information systems (GIS), remote sensing satellite imagery, geospatial techniques, and spatial statistics provide new methodologies and solutions to analyze the epidemiological and ecological context of malaria and other infectious diseases [29,30]. Recent advances in local spatial statistics [31-33] have led to a growing interest in the detection of disease clusters or 'hot spots', for public health surveillance and for improving our understanding of the disease etiology and the pathogenesis of epidemics such as malaria. While global spatial autocorrelation statistics such as the global Moran's I [34] describes the overall spatial dependence of malaria over the entire region, local spatial autocorrelation statistics such as the local Moran's I [32] and G and G* [31] are useful in identifying local patterns or hot spots.

The present study aims to examine spatial patterns or clusters of malaria distribution using village level malaria incidence data. It seeks to identify malaria "hotspot" villages and produce "hotspot" maps in different periods of observation. In addition, it describes the temporal incidence of malaria by sex and age. The ultimate objective of the study is to build spatial models of malaria risk for predicting malaria epidemics across a region or state. The findings of this study can guide policy makers and managers at different administrative levels to make evidence-based policy decisions.

## Methods

### The study area

**Figure 1:**
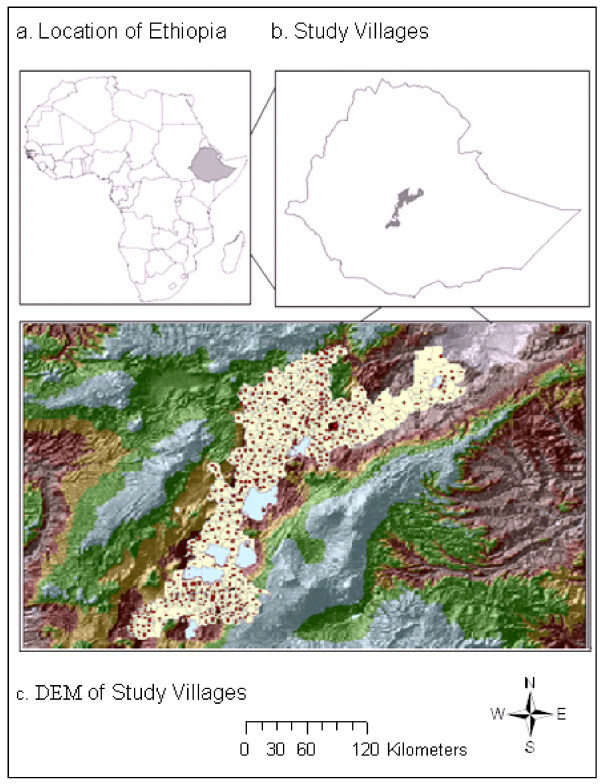
**Location of Ethiopia and the study villages, digital elevation model (DEM) showing elevation and terrain of the villages in East Shoa, Ethiopia (a-c)**.

The study was conducted in East Shoa Zone (7.08°–9.11° N, 38.11°–40.02° E), located in the Rift Valley area of Central Ethiopia (Figure 1 – top right panel). East Shoa Zone covers about 200,000 km^2^, with elevation ranging from about 902 to 2846 m (mean 1830.4 m) above sea level (Figure 1 – lower panel). Average annual rainfall is 884 mm (range 553–1126 mm), 70% of which occurs from June to September (long rainy season). Average minimum and maximum temperatures are 11.3 and 25.9°c respectively. The area comprises numerous rivers and streams that drain into the Awash River and the lakes located in the Rift Valley. Landuse consists of farmlands interspersed by patches of forests and scattered trees, pastures, shrubs, wetlands and lakes. The economy is largely based on rural subsistence-agriculture. However, a number of irrigated farms and other agro-industrial activities, such as sugarcane, maize and cotton plantations, fruits and vegetables, and horticulture also exist along the Awash River system.

East Shoa is administratively divided into 12 districts and 543 localities known as *Kebeles *(Figure 1 – lower panel) with an estimated total population of 2.3 million in 2004 [35]. There are 103 health stations and health posts, 14 health centers, 6 specialized malaria laboratory and treatment centers, and 6 hospitals in East Shoa. Malaria transmission in this region, as is in similar regions of the country, is seasonal and unstable, characterized by frequent epidemics with peaks from September to December and from April and June. About 60% of the malaria is due to *P. falciparum *infections while the remaining 40% is due to *P*.*vivax *infections [36].

#### Data types and sources

##### Malaria morbidity data

We collected individual-level daily malaria morbidity data from six specialized Malaria Diagnosis and Treatment Centers (MDTC) between September 2002 and August 2006 in East Shoa. Patients self-presenting for ambulatory care are normally diagnosed and given antimalarial treatment service free of charge. Those who are negative for malaria are referred to the nearby health center or hospital for further investigation. Information related to each subject's characteristics and diagnosis outcome is recorded into a Microsoft Access database established in these laboratories. Data collected include patient identification number, date of examination, sex, age, place of residence, travel history, presence or absence of malaria, and malaria parasite species. Subjects who had malaria infections were treated in accordance with the national treatment guidelines. A trained laboratory technician at each laboratory (within the existing system) recorded and entered data for each individual patient into a (Microsoft Access) database on a daily basis, resulting in a complete patient database.

##### Geographic information system (GIS)

Trained field health workers, using global positioning system (GPS) units, recorded data on geo-reference coordinates (villages, kebeles, health facilities and schools), and altitude for each location. A structured village mapping survey questionnaire was used to record the location information and other relevant environmental and demographic data. Kebele, district and Zonal boundaries and polygon shapes for the entire study area and other geographic features were obtained from the Ethiopian Mapping Authority (EMA) and the WHO/HealthMapper. In addition, the centroid for each village was extracted using ArcGIS 9.2 software to verify data recorded using hand-held GPS units. Digital Elevation Models (DEM) data for the study area were also downloaded from the website  and extracted using spatial analyst tools in ArcGIS 9.2. All the relevant shape geographical features were re-projected to WGS_1984_UTM_Zone_37N to integrate the dataset into one geodatabase, a data structure used in ArcGIS.

#### Data preparation and analysis

Data recording errors related to village names in the malaria database were checked against the records in the geodatabase. All records were given a unique identification code. All individual-level patient data were then cross-linked to villages by unique identification codes to facilitate both the patient level and village level analysis. Data were then aggregated by sex, age and village to describe the characteristics of the distribution of malaria incidence among the study subjects and among the 543 villages. The temporal variation in malaria distribution over the four year study period was also noted by parasite species and month.

Population estimates for the entire East Shoa Zone by village, sex, and age for the years 2002 to 2006 is calculated based on the 1994 national census and the mean annual population growth rate of 2.96% [35]. People living up to 2200 m altitude are considered to be at risk of malaria and nearly all people in East Shoa are treated as an at risk population.

A distinction is often made between prevalence and incidence of a disease. Prevalence is a measure of the total number of cases of disease in a population at a point in time, while incidence rate is the occurrence of new cases of disease (incident number) in a population divided by the person-time over a specified period [37]. Thus, prevalence indicates the magnitude of disease burden whereas incidence conveys information about the risk of contracting malaria. In the present study, the measurement of incidence is complicated by changes in the population at risk, since sometimes the same person may report more than once during a month to the malaria clinic. Each episode of malaria roughly lasts for a week and utmost for one month in the presence of recrudescence. In these circumstances, the definition of incidence is usually restricted to the first event reported in that month. Once a person is classified as a malaria case, he or she is no longer liable to become a new case within the same month. Beyond one month, the person reporting and testing positive at a clinic is considered a new case. Therefore, the incidence density (ID) of malaria was calculated by relating the numbers of new cases to the person years at risk, calculated by adding together the periods during which each individual member of the population is at risk during the measurement period [37]. ID is defined as: *Number of new cases/Total person years at risk*.

The malaria incidence density per 1000 person-years is calculated from the total number of new cases occurring in each age cohort of females and males divided by the total person-years and then multiplied by 1000. We assumed a Poisson distribution given that the incidence in this study is count data. We used the Generalized linear model [34] (GENMOD) Procedure for Poisson regression analysis in SAS using the log link function to assess the significance of variability in the incidence density ratios (IDRs) by sex and age.

Log [E(y)/person-years] = Intercept + Sex + Age + Age^2 ^+ Sex*Age + Sex*Age^2^

Where E(y) is equal to the expected number of malaria cases. A quadratic model of age raised to the second power best fit the age variable in this dataset.

#### Spatial statistical analyses

Clustering in malaria incidence was analyzed using both global and local spatial autocorrelation statistics over four specific study periods (2002/2003–2005/2006). The spatial weight matrices were defined at varying distance lags (1 km, 2 km, 3 km, 5 km, 10 km, 15 km, 20 km, and 25 km). Two assumptions guided the choice of the threshold distances: i) the effective flight range of malaria vector mosquitoes; and ii) the estimated travel distance of patients' from home to the laboratories. For this analysis, we selected 353 villages out of a total of 543 villages since they had a consistent dataset over the entire study period, allowing comparison between years.

First, the global Moran's I test statistic [32] was computed to test the null hypothesis (Ho) of no significant clustering of malaria incidence in the entire study region (α = 0.05). The test was repeated using permutations of 100, to validate the consistency of results [34]. Second, we used local Moran's I test statistics [32] to examine the presence or absence of local spatial autocorrelation using the number of malaria cases between pairs of villages at varying distance lags for each of the four specific time periods, 2002/03–2005/06 (September-August). In this context, Local Moran's I statistics is defined as follows:

$$I_{i}(d)=(x_{i}-\bar{x})\sum_{j=1}^{n}W_{ij}(d)(x_{j}-\bar{x}),\;j\neq i$$

Where $\bar{x}$ is the average number of malaria cases in villages, x_*i *_and x_*j *_are the number of malaria cases at village *i *and *j*, respectively, and w_ij _is the spatial weight matrix based on the defined distance lags (in km) between village *i *and village *j *(where *W*_*ij*_(d) = 1 if the distance between village *i *and *j *is within *d*; otherwise *W*_*ij*_(d) = 0). The mean global and local Moran's I values were plotted as a function of distance lags (km) for each specific time period. In this case, large and positive mean Moran's I values (higher than zero), indicate presence of significant clustering while negative values (less than zero) indicate dissimilar or variable patterns, and values equal to zero indicate presence of a random pattern.

Third, local G_i_* statistics [31] were calculated for each village based on the spatial weights using different threshold distances (d) as described above.

$$G_{i}^{*}(d)=\sum_{j}W_{ij}(d)x_{j}/\sum_{j}x_{j}$$

Where, w_*ij *_is a spatial weight matrix at a given distance lag in kilometers (d) (w_*ij*_(d)) is 1 when the distance from village *j *to i is within *d*, otherwise w_*ij*_(*d*) is 0). The presence of local clustering of malaria cases in the study villages (hotspot areas) was determined using Z-score values. A high and positive Z score value, >1.96, indicate that the village *i *is surrounded by relatively high malaria incidence villages, whereas a high but negative Z-score value indicates that the village *i *is surrounded by relatively low (cold spot) malaria incidence villages. Z-score values ≥ -1.96 and ≤ 1.96 indicates presence of a variable or random distribution. The results for each time period help in visualizing the temporal shifts in the location of hotspots in malaria over the four time periods.

## Results

### Characteristics of study subjects

A total of 297,046 outpatients were clinically (microscopically) examined for the presence or absence of malaria infections at the six malaria laboratory and treatment centers between September 2002 and August 2006. There were 221 observations (0.05%) with missing values either in the response or explanatory variables that were discarded from the analysis, resulting in a complete dataset of 296,825 patients. The median age of the study subjects was 19 years (range: <1–100 years). The age and sex distribution of the study subjects attending the six malaria laboratory centers are comparable with the last national population census data [35].

### Distribution of malaria incidence by village

Table 1 shows the distribution of malaria cases amongst the 497 villages or kebeles out of a total of 543. There are no outpatients coming from the remaining 46 villages. There are wide variations in the distribution and proportion of malaria incidence between kebeles. The mean number of malaria cases is 165 while the mean of the *P. falciparum *is 99 and *P. vivax *is 68. However, the median age of malaria patients shows no significant variation across villages and is around 17–19 years.

**Table 1:** The distribution of the incidence of confirmed malaria cases (all types, *P. falciparum *and *P. vivax*) per village in 497 villages in East Shoa, Central Ethiopia

**Variables**	**Mean**	**SD**	**Median**	**Min**	**Max**
No. of examined outpatients	597	1625	63	1	20021
No. of malaria cases (all types)	165	467	19	0	5873
No. of *P. falciparum *cases	99	325	12	0	3919
No. of *P. vivax *cases	68	185	7	0	1999
No. of *P. falciparum *gametocytes	13	41	2	0	547
No. of *P. vivax *gametocytes	0.1	0.6	0	0	10
Proportion of malaria cases	0.276	0.099	0.254	0	1.00
Proportion of *P. falciparum *cases	0.165	0.094	0.143	0	1.00
Proportion of *P. vivax *cases	0.113	0.056	0.1	0	1.00
Proportion of *P. falciparum *cases with gametocytes	0.022	0.016	0.017	0	1.00
Proportion of *P. vivax *cases with gametocytes	0.002	0.001	0	0	1.00
Median age of malaria cases (all types) (yrs)	17.9	2.97	18	1	44
Median age of *P. falciparum *cases	18.5	2.98	19	1	68
Median age of *P. vivax *cases	16.7	3.74	17	1	52
Median age of *P. falciparum gametocyte carriers*	19	5.03	20	1	68
Median age *P. vivax *gametocyte carriers	13.2	9.14	9.5	0.5	30

### The distribution of malaria cases by sex and age

**Figure 2:**
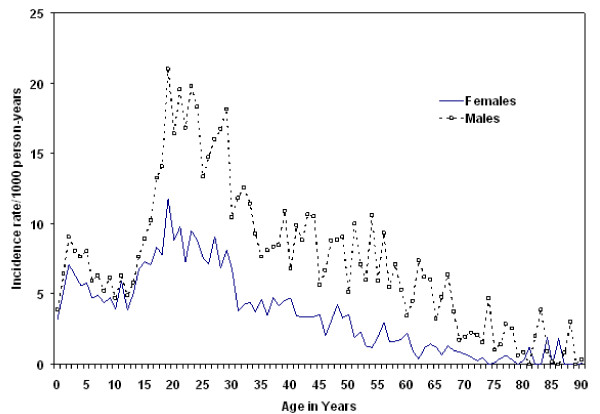
**Malaria incidence rate (per 1000 person-years) by age and gender in East Shoa, Ethiopia**.

Of the total 296,825 tested outpatients, 81,883 (about 28%) had confirmed malaria infections. Malaria was prevalent in about 30% of the total examined males and 25% of the total examined females. Overall, males comprise 61.1% of the total malaria cases. About 60% of the total malaria cases exhibited *P. falciparum *infections while the remaining 40% exhibited *P. vivax *infection. Mixed infections of both falciparum and vivax malaria were found in less than 0.1% of the cases. Figure 2 shows the incidence rate of malaria per 1000 person-years (Y-axis) in males and females by age (X-axis). In areas with unstable transmission such as East Shoa, the entire population has no immunity and is at risk for malaria. Hence one would expect the line graphs to be flat since all age groups and both sexes are at a similar risk. But that is not the case as shown in Figure 2. First, the cumulative incidence rates are higher in males than females in all ages (except around 80–88 years) indicating that males are at a higher risk. Second, the line graphs show the presence of a non-uniform relationship between the cumulative malaria incidence rate and age in both sexes, with a peak between 15 and 30 years of age, notably higher for males. These results are further supported by analysis in Table 2.

**Table 2:** The association of malaria incidence rate per person-year with sex, age, and sex-age interaction using a Poisson regression model

			Wald 95% Confidence Limits		
			
Parameter	DF	Estimates	Lower	Upper	P-Value
Intercept	1	0.0050	0.0049	0.0051	<0.0001
Age	1	1.0657	1.0636	1.0677	<0.0001
Age-square	1	0.9989	0.9988	0.9989	<0.0001
Females	1	0.8648	0.8357	0.8949	<0.0001
Males	0	1.0000	1.0000	1.0000	.
Age*females	1	0.9811	0.9780	0.9841	<0.0001
Age*males	0	1.0000	1.0000	1.0000	.
Age-square*females	1	1.0001	1.0000	1.0002	0.0009
Age-square*males	0	1.0000	1.0000	1.0000	.
Scale	0	2.7183	2.7183	2.7183	

Poisson regression model results shown in Table 2 indicate the significance of age and sex on cumulative malaria incidence rates. A significant increase in the incidence rate is observed per one-year increase in age (1.07, 95% CI [1.06, 1.07], P =< 0.0001). The malaria incidence rate in females is found to be significantly lower than in males (0.86, 95% CI [0.84, 0.89], P =< 0.0001). The results also indicate a statistically significant interaction between age and sex (0.98, 95% CI [0.978, 0.984], P = 0.0001).

### Temporal variation in the distribution of the incidence of malaria cases

**Figure 3:**
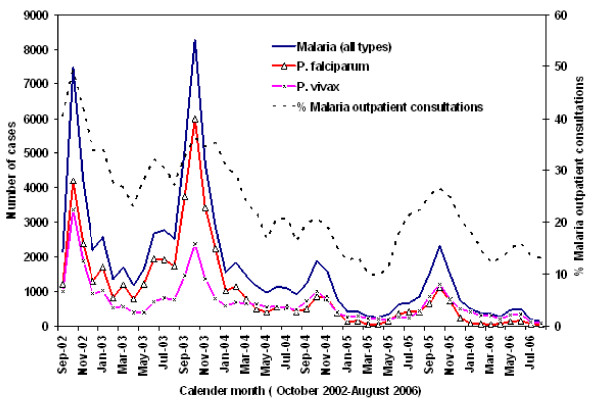
**Monthly distribution of malaria incidence from six laboratory centers, East Shoa, Ethiopia**.

Figure 3 presents the temporal variations in the monthly number and relative proportion of malaria cases out of the total over a period of four contiguous years (September 2002–August 2006). The average (standard deviations) number of malaria cases per month is about 1706 (1729 standard deviations). Higher malaria incidence, above normal, is observed from September 2002 to December 2003, with a peak during the month of October. Most of the malaria incidence, 25% of the annual total number of incident malaria cases, is observed in October and a great majority of this is due to *P. falciparum *infections. However, there is a general decreasing trend, characterized by a similar seasonal pattern, in the number and relative percentage of incident malaria cases beginning in January 2004. The frequency *P. falciparum *gametocyte carriers are shown to be high during 2002/2003.

### Spatial distribution of malaria incidence by village

**Figure 4:**
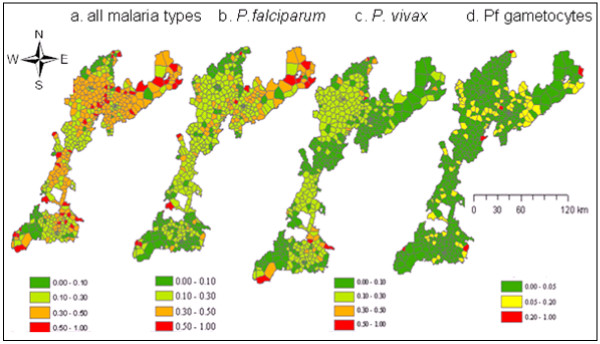
**Spatial distribution of the malaria incidence (all malaria types, *P. falciparum*, *P. vivax*, and *P. falciparum *gametocytes) in East Shoa, Ethiopia (a-d)**.

Figure 4 shows the spatial distribution of the proportion of malaria cases (all malaria types, the two malaria parasite species) over the four-year study period. Panel 1 shows a higher incidence of all malaria in the northern villages as well as some pockets in the south. Panel 2 shows a higher proportion of *P. falciparum *cases in low altitude areas while Panel 3 shows *P. vivax *in high altitude areas. Villages with high (above 5%) gametocyte carriers are shown in red and yellow colors in Panel 4. These maps suggest that the spatial pattern in the incidence of malaria could be correlated to critical determining factors, such as elevation, rainfall and landuse

### Global and local patterns of the incidence of malaria at village level

#### Moran's I test statistics for global spatial autocorrelation

**Figure 5:**
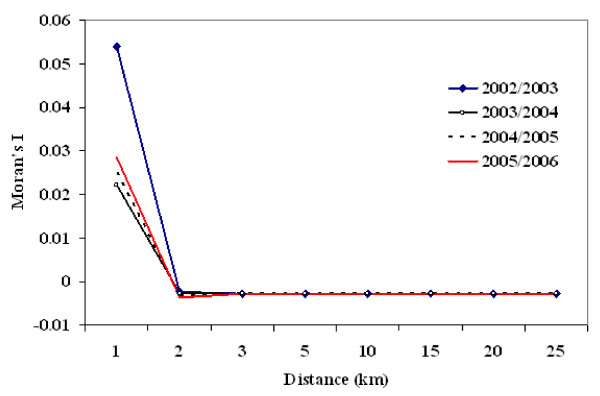
**Mean Moran's I values for global spatial autocorrelation for malaria incidence at varying distance lags (in km), East Shoa, Ethiopia**.

First, global spatial statistics are estimated using the Moran's I measure. The test results indicate the presence of significant global spatial autocorrelation for the incidence of malaria (all malaria types, *P. falciparum *and *P. vivax*) at 1 km threshold distance lag for all villages. However, these global Moran's I test statistics are not significant at distances greater than 2 km (Figure 5). The test results are similar for both *P. falciparum *and *P. vivax *cases (data not shown here). However, these global results suggest that there are strong local patterns in the distribution of malaria that need to be further explored using local spatial statistics.

#### Local Moran's I test statistics for local spatial autocorrelation of malaria incidence

**Figure 6:**
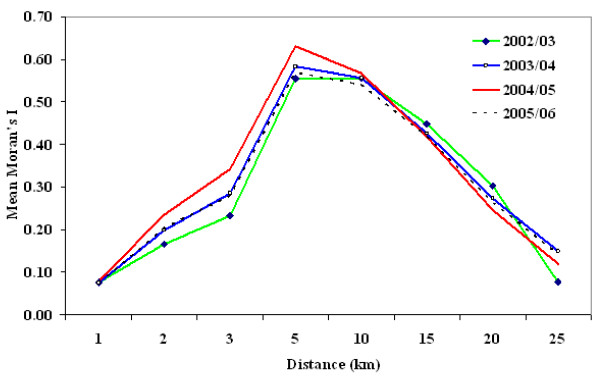
**Mean Moran's I values for local spatial autocorrelation for malaria incidence at varying distance lags (in km), East Shoa, Ethiopia**.

Local Moran's I statistics are estimated for the incidence of malaria (all types,*P. falciparum *and *P. vivax*) for the four specific time periods 2002/03–2005/06 (September-August). Figure 6 shows graphical plots of the overall mean local Moran's I value as a function of distance (in km). Highest mean local Moran's I values are observed at 5 and 10 km distance lags, although the overall mean test results indicate no statistically significant local clustering of malaria incidence at the 0.05 level. However, statistically significant local clustering of malaria incidence is detected in four villages (Basaku Ilala, Meja Karsa, Aga, and Gonde Gurati) out of a total of 353 villages in 2002/03, 2003/04, 2004/05 and 2005/06, respectively, at 5 km distance lags. Similarly, significant local clustering of malaria incidence is detected in the following three villages, namely Mechafera, Faji Sole, and Aleche Hare Bate, in 2002/03, 2003/04, 2004/05 and 2005/06 at 10 km respectively. Similar local clustering patterns are also observed for each of the two parasite species over the four time periods. These local statistics reveal distinct spatial patterns.

To better visualize Local Moran's I values, we performed a spatial interpolation procedure to transform our village (point) data into area based maps using Thiessen polygon interpolation.

**Figure 7:**
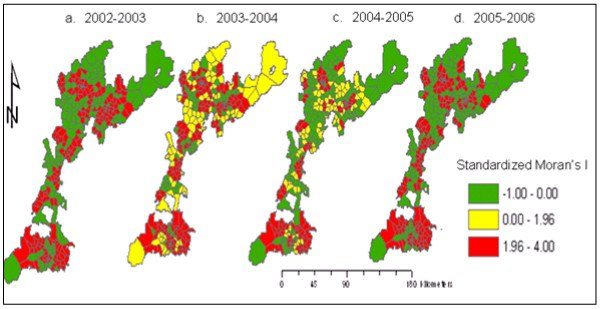
**Standardized local Moran's I values showing clustering of malaria incidence in 353 villages in East Shoa, Ethiopia (a-d)**.

Figure 7 shows the spatio-temporal distribution of standardized local Moran's I for malaria incidence over the four time periods. The maps show villages with significant clustering, Moran's I values of greater than 1.96 Z score values, in red. There is more significant local clustering in the 2002–03 and 2005–06 time periods, with clusters in northern and southern villages.

#### Local G* test statistics results for malaria incidence

**Figure 8:**
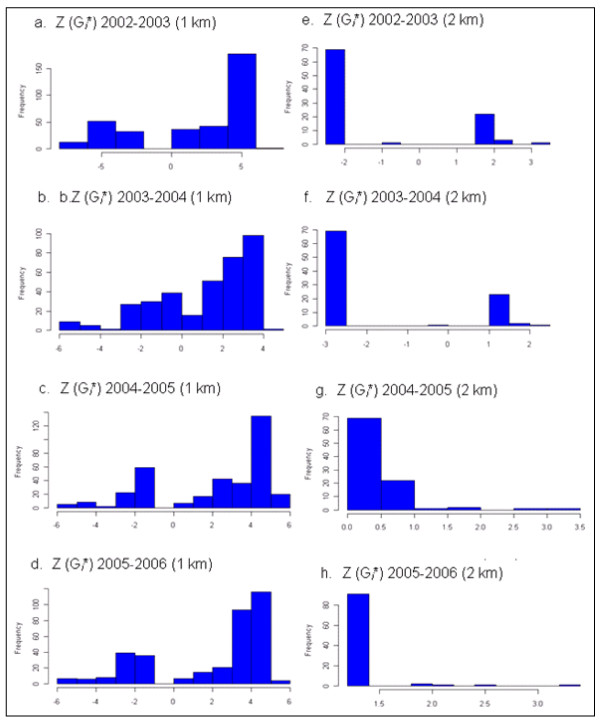
Standardized local Gi* values of spatial association for malaria incidence at 1 km (a-d) and 2 km (e-h) distance lags, East Shoa, Ethiopia.

Local spatial statistics G* is calculated for each village to detect the presence of significant local clustering of malaria incidence over the four time periods at different threshold distances. Figure 8 shows the frequency distribution of villages against standardized (G*) values, indicating the presence of malaria hotspots at 1–2 km distance lags. However, the number of villages with significant local clustering decreases at 2 km distance lags. No malaria hotspots are detected beyond 2 km distance lags. The local G* test statistics for each parasite species, *P. falciparum *and *P. vivax*, display similar results.

## Discussion

This study describes the non-spatial and spatial pattern of malaria distribution in areas of seasonal unstable transmission using routinely collected individual-level patient morbidity data from health care facilities. Our findings are presented below.

First, malaria transmission varies due to gender and age. The high incidence rate observed in males as compared with females might be due to different exposure rates or other behavioral risk factors. Males in the age group 15–35 years may be at risk due to greater mobility and work related activity in the fields and risky areas. Females and children tend to stay indoors and travel less, and may use mosquito netting and other precautions against exposure. The presence of interaction between sex and age further highlights the differences in exposure to the risk of infection. It is important to point out that the relative proportion of males and females attending the malaria laboratory centers in the present study are comparable to the national census figure, indicating an absence of differential use of these health care services across groups. Malaria affects all age groups of the population in areas with unstable malaria transmission [4,38,39]. However, the observed quadratic relationship between the incidence rate of malaria and age remains to be understood. Hence, further investigation is warranted to determine the underlying differences in exposure or behavioral risk factors to prevent incidence spikes in certain age groups and in men.

There are temporal variations in the incidence of malaria that may be related to variations in climate [12,13], and other local environmental risk factors [24,40]. Most malaria occurs during September and December with peaks during the month of October, immediately after the main rainy season. But there are significant inter-annual variations, with elevated incidence in 2002–2004 and reduced incidence in 2005–2006. In 2003, unusually severe and intense malaria epidemics were reported in several other highland areas (1500–2500 m) in four regions in the country [8,41]. These epidemics have partly been associated with climate abnormalities (lower rainfall and warmer temperature) linked to El Nino events, and partly due to an increase and spread of antimalarial drug resistant-falciparum malaria, and the neglect/breakdown of vector control operations [8,11-13]. The relative contribution of these risk factors needs to be fully investigated.

Similar severe malaria epidemics, associated with climate abnormalities, have also been reported to occur in several Eastern and Southern African countries [41]. On the other hand, the decreasing trend in malaria incidence after 2003/2004 is perhaps due to unfavorable climatic conditions coupled with a decrease in the proportions of susceptible populations, and the likely impact of control measures intensively implemented soon after 2003/2004. *P. falciparum *generally predominates *P. vivax *during the period of increased transmission or epidemic years, while *P. vivax *dominates during low transmission or non-epidemic years. Such temporal variation in the relative frequency of the two parasite species might be related to a decrease in temperature and the effect of antimalarial drugs used. In addition, high *Pfalciparum*. gametocyte carriages in the population are observed during these peak periods. Such high carriage rates might be related to delay or ineffective treatment. This factor also plays a significant role in increasing the transmission rates.

None of the global Moran's I test statistic shows significant clustering above 1 km. The global Moran's I test is likely to be biased towards the null hypothesis since small variations in malaria incidence in villages or higher variations at few villages are not effectively captured by an overall global measure of spatial association. A climate-altitude based gradient of malaria transmission is not observed in the entire study region, although altitude in these villages varies form 940 m to 2800 m and climate can be expected to vary in relation to altitude. The lack of a climate-altitude based gradient of malaria transmission suggests that local environmental and socioeconomic factors that operate at smaller spatial scales might be more critical [42-46]. Further investigation is warranted to understand the effect of climatic and topographic factors on malaria incidence in the study region.

Significant local clustering of malaria incidence occurs between pairs of villages within 1–10 km distance lags with high values observed at 5 and 10 km for local Moran's and at 1–2 km for local G* statistics. The local test statistics Moran's I and G* results suggest that there are significant clusters or hotspots of malaria incidence. Local risk factors such as proximity to water bodies, topography, access to health facilities, control measures and others might all be important in explaining the observed local clustering of malaria incidence. Thus, our study suggests that local spatial variations at the village level are more useful and add value in prevention programs. Certain clusters of villages have persistent increases in malaria incidence and thus anti-malarial interventions such as insecticide treated bed nets (ITNs) and indoor residual spraying of houses (IRS) can be targeted to those persistent hot spots, eliminating wasteful spending.

The surveillance data analyzed in this study has obvious limitations related to coverage and completeness, and thus can underestimate the actual malaria incidence rate in the population, especially in some remote locations. Nonetheless, this study illustrates the importance of surveillance data in identifying malaria hotspots, which cannot be accomplished using population surveys.

Findings from this study can be used for designing early warning decision support systems that enable the efficient and timely spatial targeting of preventive and control measures against the disease. The study has practical utility in making timely risk maps that can be used to communicate disease risk easily. This study is part of an ongoing research project undertaken by the authors.

## Competing interests

The authors declare that they have no competing interests.

## Authors' contributions

The author AK was involved in the conceptualization, research design, data collection, implementation and manuscript writing. SG contributed in the design and analysis and manuscript writing. AT and DO were involved in the conceptualizing, research design, field data collection and contributed in reviewing the manuscript. HP contributed in data analysis and preparation of the manuscript.

## Disclaimer

This manuscript was completed by Asnakew Kebede in partial fulfillment of the Master of Arts degree at the Department of Geography and Environment, Boston University. The opinions of the authors expressed in the manuscript do not reflect the views of the authors' affiliated organizations.
